# Effect of Metal Artifacts on Detection of Vertical Root Fractures Using Two Cone Beam Computed Tomography Systems

**DOI:** 10.7508/iej.2015.03.010

**Published:** 2015-07-01

**Authors:** Yaser Safi, Mohammad Mehdi Aghdasi, Fatemeh Ezoddini-Ardakani, Samira Beiraghi, Zahra Vasegh

**Affiliations:** a *Department of Oral and Maxillofacial Radiology, Dental School, Shahid Beheshti University of Medical Sciences, Tehran, Iran*; b *Department Oral and Maxillofacial Radiology, Dental School, Shahid Sadoughi University of Medical Sciences, Yazd, Iran*; c *Dentist, Tehran, Iran*

**Keywords:** Cast Post, Root Fracture, CBCT, Cone-Beam Computed Tomography, Vertical Root Fracture

## Abstract

**Introduction:**

Vertical root fracture (VRF) is common in endodontically treated teeth. Conventional and digital radiographies have limitations for detection of VRFs. Cone-beam computed tomography (CBCT) offers greater detection accuracy of VRFs in comparison with conventional radiography. This study compared the effects of metal artifacts on detection of VRFs by using two CBCT systems.

**Methods and Materials:**

Eighty extracted premolars were selected and sectioned at the level of the cemento enamel junction (CEJ). After preparation, root canals were filled with gutta-percha. Subsequently, two thirds of the root fillings were removed for post space preparation and a custom-made post was cemented into each canal. The teeth were randomly divided into two groups (*n*=40). In the test group, root fracture was created with Instron universal testing machine. The control teeth remained intact. CBCT scans of all teeth were obtained with either New Tom VGI or Soredex Scanora 3D. Three observers analyzed the images for detection of VRF. The sensitivity, specificity, positive predictive value (PPV) and negative predictive value (NPV) for VRF detection and percentage of probable cases were calculated for each imaging system and compared using non-parametric tests considering the non-normal distribution of data. The inter-observer reproducibility was calculated using the weighted kappa coefficient.

**Results:**

There were no statistically significant differences in sensitivity, specificity, PPV and NPV between the two CBCT systems.

**Conclusion:**

The effect of metal artifacts on VRF detection was not significantly different between the two CBCT systems.

## Introduction

Vertical root fracture (VRF) is among the most common causes of endodontic treatment failure. Detection of root fracture, particularly VRFs, is clinically challenging [1-3]. From the coronal aspect, VRFs initiate at the root canal wall and propagate towards the root surface [4]. VRFs may involve one (incomplete) or both sides (complete) of the root. In sagittal aspect, VRFs may also be classified as complete or incomplete involving some part or the entire cervico-apical length of the root [5, 6]. The prevalence of VRFs in endodontically treated teeth varies from 3.7 to 30.8% [7-10]. Maxillary and mandibular premolars and the mesial root of the mandibular molars are more susceptible to VRFs [11, 12]. 

Detection of VRFs is much more difficult than crown fractures since transillumination, bite test and using dyes cannot be performed as easy as crown fractures and require surgical exposure of the root [13]. A comprehensive dental history in conjunction with clinical and radiographic signs and symptoms such as history of pain, sinus tract close to the gingival margin, abscess, sensitivity to palpation or percussion, deep bony lesions and periapical or lateral radiolucencies related to the root can provide valuable information suggesting the presence or absence of VRFs [14-17]. A prompt decision regarding extraction to cease the process of rapid bone loss is necessary when the fracture is exposed to the oral environment. Moreover, due to the poor prognosis of VRFs, a reliable diagnostic technique is necessarily [18].

Intraoral digital imaging systems have been used for more than 20 years as an alternative to the conventional film-based systems [12, 19]. However, both digital and conventional radiographic techniques have low sensitivity for detection of VRFs [20, 21]. This limitation is attributed to several factors including the superimposition of the adjacent anatomical structures, the x-ray beam not being parallel to the fracture line and representing a two-dimensional (2D) image of a three-dimensional (3D) structure [13, 22]. Inability of the non-invasive, conventional imaging techniques for accurate detection of VRFs emphasizes on the importance of advanced alternative imaging techniques to enhance the diagnosis of such defects [23]. Recently, cone-beam computed tomography (CBCT) has gained increasing popularity in dentistry for diagnostic, treatment planning and follow-up purposes [24]. Data obtained from CBCT images without overlapping of the adjacent structures define the problem more accurately and enable tailor-made treatment planning for VRFs [3, 15, 16, 25]. 

However, it should be noted that in about 90% of the teeth with VRFs, the root canals are filled with gutta-percha while intra-canal posts are present in approximately 61.7% of cases [16]. These materials cause streak-like artifacts in CBCT images and significantly decrease the diagnostic accuracy, since the dark streaks may be mistaken for fractures and the light streaks may mask the actual fracture lines and account for the cases of false positive and false negative results. The magnitude of reduction in the diagnostic accuracy of imaging systems due to root canal filling materials and intra-canal post artifacts is variable in different studies. Several factors affect the level of artifacts and the magnitude of reduction in diagnostic accuracy of images [25-27]. The present study, tried to assess the effect of intracanal post on diagnostic accuracy of two CBCT systems in terms of detecting VRFs.

## Materials and Methods

This *in vitro* study was conducted on 80, single-rooted human premolars without any root fracture. The teeth were selected irrespective of the patients’ age and gender or extraction reason. Prior to the experiment, the teeth were cleaned from the soft tissue residues and debris. The teeth were immersed in 0.05% sodium hypochlorite solution to eliminate organic residues and were then stored in saline solution to prevent dehydration. The crowns were separated from the roots at the level of the cemento enamel junction (CEJ) using a metal disc [26]. The coronal part of each root was flared using #1-3 Gates Glidden drills (Dentsply, Maillefer, Ballaigues, Switzerland) and the apical section was filed with hand K-files up to #50 up to 1 mm shorter than the apex. The root canals were then filled with gutta-percha (AriaDent, Tehran, Iran) and sealer (AH-26, Dentsply, De Trey, Konstanz, Germany). 

One week later, the root fillings in the coronal 2/3 of the roots were removed using #2 and 3 Piezo drills (Dentsply, Maillefer, Ballaigues, Switzerland) for post space preparation. Post pattern was made using Duralay acrylic resin (Reliance Dental Mfg. Co., Worth, IL, USA) and custom-made posts were casted with nickel chromium alloy. One layer of wax was wrapped around the roots and the teeth were mounted in acrylic blocks. Then, 40 teeth were randomly chosen for induction of root fracture. To create fracture, brass pins were placed in the root canals and the fracture was artificially created using Instron Universal Testing Machine (Z010, Zwick GmbH, Ulm, Germany) which applied an increasing load on the pin until fracture occured and then the load was immediately stopped as shown by the diagram displayed on the system monitor. No fracture was created in the remaining 40 teeth and they were considered as controls. 

For gold standard determination after imaging, all specimens were removed from the acrylic resin and stained with 1% methylene blue dye. The teeth were observed under a magnifier and presence of fracture in the test group and absence of fracture in controls was ensured [28]. All specimens were kept hydrated during the study period and were removed from the saline solution only for the fabrication of post, induction of VRF and imaging. 

**Figure 1:**
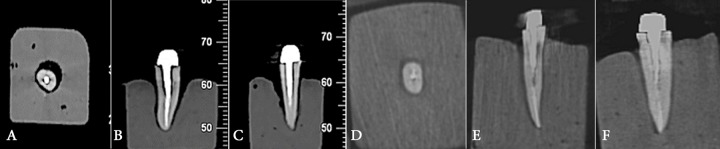
CBCT scans of teeth in New tom VGI system; *A)* Axial, *B)* Coronal, *C) *Sagittal and Scanora 3D; *D) *Axial, *E)* Coronal, *F)* Axial

To obtain CBCT scans, the teeth were randomly divided into 8 groups of 10 and arranged to form an arc on the chin rest of CBCT New Tom VGI system (Quantitative Radiology, Verona, Italy) (with 0.2 mm focal spot, rotary anode and 20×25 cm sensor) in 12×8 cm field of view (FOV), and then in Scanora 3D CBCT system (Soredex, Helsinki, Finland) (with 0.5 mm focal spot size, fixed anode and 12.5×12.5 cm sensor size) with 10×7.5 cm FOV; the exposure setting in both systems included 0.2 mm voxel size at 90-110 kVp and 12.5 mA (Figure 1). The average FOV of systems was applied so they could completely cover the dental arches in this study.

Three blinded oral and maxillofacial radiologists evaluated the scans for presence of VRFs. The observers were allowed to adjust the contrast and brightness of the images as desired. No time limit was set for evaluation of images. All images were observed on LG Flatron W1752s LCD monitor with 1440×90 dpi display resolution. The coronal and sagittal sections referred to in this study were not the actual coronal and sagittal sections of the respective teeth because the teeth were positioned in the form of an arc on the chin rest. After observing the CBCT scans in the three dimensions, the observers graded their responses as follows: *grade 1*: definitely no fracture; *grade 2:* probably no fracture; *grade 3:* not sure about the presence or absence of fracture; *grade 4:* probable fracture and *grade 5:* definite fracture [22].

In the current study, the observers were allowed to enhance the images and adjust the magnification of section in CBCT scans of the two systems and could use all the enhancement filters whenever required. The observers were first instructed on how to use the software of the two systems and then used different filters to change the contrast, resolution, magnification, *etc.,* based on their diagnostic experience. Considering the scale used for reporting the observers’ diagnosis, the results were described as absolute (deterministic) and probabilistic sensitivity and specificity. Absolute sensitivity and specificity describe the observers’ clinical opinion regarding definite presence or absence of VRFs and were specified as definite diagnosis of VRFs and intact teeth, respectively. 

Total sensitivity and specificity also included their opinion regarding the possible presence or absence of VRFs and were calculated as definitely and probable diagnosis of real VRFs (total sensitivity) and fracture-free teeth (total specificity). The undetectable cases for which the observers could not definitely or probably define the presence or absence of fracture lines were not excluded from the analysis. They were accounted in all of the cases that really had fracture or not according to the gold standard. The positive predictive value (PPV) was defined as number of true positives/number of total positive calls and the negative predictive value (NPV) was defined as number of true negatives/number of total negative calls.

Statistical analysis was carried out using statistical package for social sciences (SPSS software, version 19, SPSS, Chicago, IL, USA). The sensitivity, specificity, PPV and NPV for detection of VRFs and percentage of probable cases were calculated for each imaging system and compared using non-parametric tests considering the non-normal distribution of data. The inter-observer reproducibility was calculated using weighted kappa coefficient.

## Results

The overall grades for both groups are presented in Tables 1 and 2. The absolute sensitivity values of the two CBCT systems were very close and the difference between them was not statistically significant (*P*=1). The same results were obtained for total sensitivity values (*P*=0.7), absolute specificity values (*P*=1) and total specificity values (*P*=0.7) (Table 1). The deterministic PPV was not significantly different between the two CBCT systems (*P*=0.2), the total PPV (*P*=0.4), absolute NPV (*P*=0.4) and total NPV (*P*=0.7) were not significantly different between the two systems, either (Table 2). The inter-observer reproducibility of the two systems was calculated using weighted kappa coefficient, which was found to be 0.85 for New Tom VGI and 0.94 for Soredex Scanora 3D. No significant difference was found in inter-observer reproducibility between the two systems (*P*=0.087). 

**Table 1:** Absolute (A) and total (T) sensitivity and specificity values of the three observer for detection of VRFs (%)

**Imaging systems**	**Observer**	**A-Sensitivity**	**T-Sensitivity**	**A-Specificity**	**T-Specificity**
**New Tom VGI**	First	7.5	55	7.5	62.5
	Second	30	60	17.5	52.5
	Third	25	27.5	17.5	52.5
**Scanora 3D**	First	5	25	7.5	55
	Second	30	75.5	10	37.5
	Third	27.5	67.5	25	32.5

**Table 2:** Absolute (A) and total (T) positive and negative predictive values (PPV, NPV) among three observers for VRF detection (%)

**Imaging systems**	**Observer**	**A-PPV**	**T-PPV**	**A-NPV**	**T-NPV**
**New Tom VGI**	First	62.5	75	59.4	60
	Second	56.75	58.3	57.14	70.6
	Third	48.27	58.3	47.8	70.6
**Scanora 3D**	First	44	60	37.07	33.3
	Second	60	66.7	54.5	57.1
	Third	54.16	76.9	50.94	61.1

## Discussion

The purpose of this study was to examine the effect of metal artifact on diagnosis of VRFs in two CBCT systems. Definite diagnosis of VRFs is a challenge for clinicians since the clinical and radiographic signs and symptoms are not pathognomonic and mimic those of endodontic failure and periodontal lesions [29]. Indefinite diagnosis often leads to invasive, unnecessary surgical procedures or tooth extraction. Thus, prompt in time diagnosis is necessary [29, 30]. The results of the present study showed no significant difference between two CBCT systems. This is possibly due to several interfering factors such as voxel size, exposure settings, FOV, slice thickness, presence or absence of gutta-percha, type of post and particularly the type of the imaging system and the image detector. These parameters are variable in different CBCT units and different imaging protocols in the same unit, which has also been observed in the previous studies [30, 31].

The radiation dose of CBCT depends on the model of CBCT unit, and the applied protocol. Lowering the resolution, decreases the scan time and consequently the patients’ radiation dose [15]. Our results are consistent with Hassan *et al. *[31] stating that the axial section of the root is the most suitable for detection of VRFs. All three observers first viewed the axial and then the coronal and sagittal sections, which is similar to the other studies [22, 31]. Some other conclusions reported by Hassan *et al.* [31], confirm the results of the present study: flat-panel-detector CBCT systems such as Scanora 3D and New Tom VGI had less artifact, less noise, less contrast and higher resolution compared to CCD detectors.

The study by Metska *et al. *[32], confirms the validity of CBCT scanners for detection of VRFs and mentioned that the diagnostic accuracy for detection of VRFs in teeth with intracanal posts depends on the imaging system. They reported that the sensitivity, specificity and diagnostic accuracy of the 3D Accuitomo were higher than those of the New Tom 3G in terms of detecting VRFs. In the current study, type of the CBCT system definitely affected the degree of artifacts, as well.

It should be noted that ~90% of teeth with VRFs have root canal filling materials and approximately 61.7% of them have intra-canal posts [27]. These materials cause streak-like artifacts in CBCT images and significantly decrease the diagnostic accuracy, since the dark streaks may be mistaken for fractures and the light streaks may mask the actual fracture lines and account for the cases of false positive and false negative results. The magnitude of reduction in the diagnostic 

accuracy of imaging systems due to root canal filling materials and intra-canal post artifacts has been variable. According to a study by Costa *et al.* [25], the presence of a metallic post significantly reduces the specificity and sensitivity of diagnosing VRF. In our study, nickel chromium posts were used and caused substantial amounts of artifacts, similar to the other studies such as the study by Estrela *et al. *[33]. 

Evidence shows that sensitivity and specificity values for detection of VRF by CBCT systems are influenced by the amount of artifacts and are dependent on the voxel size, FOV size, presence and kind of intra-canal post, type of imaging system, type of detectors, slice thickness, VRF dimension, scanning parameters and *etc*. (19). 

Changing the thickness of the slices has no significant effect on the amount of artifacts [23]; therefore, 1-mm thick slices were used in the current study. The highest diagnostic accuracy for detection of VRFs was obtained when the distance between pieces was 0.4 mm [29]. Thus, non-displaced or hairline fractures can complicate the interpretation of results. Non-displaced fractures are usually not detectable with intraoral radiography. Such fracture lines may even remain undetected on CBCT scans due to the overlapping of anatomical structures and artifacts that can mimic or mask the fracture lines [34]. Thus, in the current study, non-displaced fractures were used to simulate the clinical setting as much as possible. 

According to the literature, *in vitro* studies have several limitations. Mora *et al. *[35] mentioned the method of creating the fracture artificially and setting the environment in which the tooth is placed. Also, the results of *in vitro* studies cannot be directly generalized to the clinical setting because in these studies, only the radiographic techniques are evaluated and clinical parameters such as the probing depth, mobility and signs and symptoms like changes of the periodontal status, bone loss, sensitivity during mastication, periapical radiolucencies, and crestal bone loss are not taken into account while they can assist in detection of VRFs [25, 36, 37]. Moreover, presence of tooth crown along with a restoration can complicate fracture detection in axial sections especially if the fracture is located in close proximity to the CEJ. In a study by Valizadeh *et al. *[38], CBCT, conventional and digital radiographic techniques were compared for detection of VRFs and the highest sensitivity (94.6%) and specificity (98.2%) values were reported for CBCT; whereas, these values were 66.7% and 76.9% for conventional radiography and 74.1% and 76.3% for digital radiography, respectively. 

**Table 3:** The mean absolute (A) and total (T) sensitivity (SNS), specificity (SPC), positive and negative predictive values (PPV, NPV

**Systems**	**T-NPV**	**A-NPV**	**T-PPV**	**A-PPV**	**T-NPV**	**T-SPC**	**A-SPC**	**T-SNS**	**A-SNS**
**New Tom VGI**	55.8±7.1	20.8±11.8	47.5±11.5	14.1±5.7	55.8±5.7	67.06±6.1	54.7±6.1	63.8±9.6	20.8±11.8
**Scanora 3D**	20.8±13.7	56±27.1	14.1±9.4	41.6±11.8	50±15.02	47.5±9.2	67.8±8.5	52.7±8.09	20.8±13.7

Regarding the effect of FOV, Felipe Costa *et al. *[25, 27] demonstrated that large FOV decreased the diagnostic accuracy for detection of VRFs irrespective of the presence or absence of intracanal posts and reported a very low inter observer agreement. Small FOVs were accurate for detection of VRFs in absence of intracanal posts but presence of post decreased this accuracy.The spatial resolution and therefore detail of a CBCT image is determined by the individual volume elements (voxels) produced in formatting the volumetric data set. The principal determinants of nominal voxel size in a CBCT image are the matrix and pixel size of the detector [39]. Detectors with smaller pixels capture fewer x-ray photons per voxel and result in more image noise. Consequently, CBCT imaging using higher resolution may be designed to use higher dosage to achieve a reasonable signal:noise ratio for improved diagnostic image quality. Although, the small voxel size was accurate for detection of VRFs in absence of intra-canal posts, presence of posts decreased the accuracy of diagnosis [30]. According to the studies by Melo *et al. *[26], Ozer *et al. *[29] and Da Silveria *et al. *[15], the proper voxel size for detection of VRFs is 0.2 mm considering low exposure doses and acceptably high diagnostic accuracy. Therefore, 0.2 mm voxel size was used in the current study.

## Conclusion

This study found no significant difference in sensitivity, specificity, negative predictive value and positive predictive value between the two studied CBCT systems. No significant difference was found in the inter-observer reproducibility between the two systems. In other words, both CBCT systems had equal diagnostic value for VRF detection.
